# Dual beta-lactam treatment: Pros and cons

**DOI:** 10.1097/j.pbj.0000000000000189

**Published:** 2022-10-24

**Authors:** Diogo Guerra, Pauline Vidal, Olivier Paccoud, Alexis Maillard, Laurene Cachera, Helga Junot, Rémy Gauzit, Jean R. Zahar, Miguel A. Abreu, Alexandre Bleibtreu

**Affiliations:** a Service des Maladies Infectieuses et Tropicales, Hôpital Pitié Salpêtrière, APHP Sorbonne Université, Paris, France; b Serviço de Doenças infecciosas do Centro Hospitalar do Porto, Porto, Portugal; c Service de Bactériologie, Hôpital Pitié Salpêtrière, APHP Sorbonne Université, Paris, France; d Pharmacie, Hôpital Pitié Salpêtrière, APHP Sorbonne Université, Paris, France; e Équipe mobile d'infectiologie, réanimation Ollier, hôpital Cochin AP-HP, Paris, France; f Infection Control Unit, Hôpital Avicenne, Groupe Hospitalier Paris Seine Saint Denis, AP-HP, Bobigny, France.

**Keywords:** antibiotics, beta-lactams, dual therapy, resistance

## Abstract

The battle against microscopic pathogens has always baffled the scientific community. Nowadays, multidrug-resistant microorganisms lead to high in-hospital mortality, increased hospital stays, and high health-related costs. Treating infections due to these high-resistance pathogens with a low number of antibiotic molecules creates the need for new strategies. Although some already think of a “postantibiotic era” with bacteriophages as the main futuristic weapon in antibacterial armament, others rethink the usage of the already existent drugs. Dual beta-lactam therapy has been used for quite some time as an empirical therapy for some severe infections such as endocarditis or meningitis. However, studies regarding the use of a beta-lactam combination stopped being made a long time ago, and it seems the scientific community has no interest in evaluating this as a treatment option. Could this strategy be applied to treat infections due to multidrug-resistant bacteria? Could this be the answer while waiting for the “postantibiotic era”? What kind of pathogens could we fight using dual beta-lactams? What are the downsides of this strategy? These are some of the questions the authors try to answer in this review. In addition, we try to convince our peers to turn once more into researching beta-lactam combinations and exploring its potential benefits.

## Introduction

Multidrug resistance (MDR) is a rising concern in today's society, especially concerning gram-negative agents. The only way to fight this threat is to invest in better infection control policies, more efficient antimicrobial use, and in the development of new molecules with novel mechanisms of action.^1^ Despite more than 10 new molecules approved by the Food and Drug Administration^2^ with expanded activity against gram-negative bacteria, the past decades have not been particularly fruitful in generating new drugs. Taking into account the rise of *Enterobacteriaceae* resistances and the shortage of new “weapons” to fight them, the scientific community has had to rely on older antibiotics, associated with weaker safety profiles, such as colistin.

An alternate means of overcoming these challenges is by combining different antimicrobial agents, thereby taking advantage of complementary mechanisms of action. One of the most studied combinations is that of a beta-lactam and an aminoglycoside, usually to enhance the bactericidal effect or to broaden the antimicrobial spectrum. Despite being widely used in clinical practice, there are still valid concerns regarding the nephrotoxicity associated with this association. Furthermore, one can argue against the need for an antibiotic spectrum overlap and the risks it could entail to bacterial ecology.

Another combination, broadly tested in the 1980s, was the so-called “dual beta-lactam therapy.” Although results were, in general, favorable to its implementation, the actual need for this type of combination was scarce because physicians could achieve a great amount of success using large doses of single drugs with expanded spectrums. Moreover, “dual beta-lactam therapy” was considered to have an antagonist effect by some authors. Since then, only a few studies have been performed. Table 1 summarizes the most important studies regarding the beta-lactam combination therapy.

**Table 1:** Articles in favor of the use of beta-lactam combination

Title	Authors	Combinations tested	Result
Amdinocillin plus cefoxitin versus cefoxitin alone in therapy of mixed soft-tissue infections (including diabetic foot infections)^6^	File Jr and Tan	MLM (amdinocillin) + CXT	Better microbiological outcomes on the combination group than cefazolin alone
Ceftriaxone versus aztreonam plus cefazolin for infections in cancer patients with adequate neutrophil counts^7^	Menichetti et al	CZN + ATR	Beta-lactam combination better than ceftriaxone alone for documented infections but worse in nondocumented infections
Comparative in vitro synergistic activity of new beta-lactam antimicrobial agents and amikacin against *Pseudomonas aeruginosa* and *Serratia marcescens*^9^	Kurtz et al	Combinations between MXL, CFZ, PIP, and TIC	Synergy between beta-lactams but less powerful than between beta-lactams and aminoglycosides
Comparative activity of cefepime, alone and in combination, against clinical isolates of *Pseudomonas aeruginosa* and *Pseudomonas cepacia* from cystic fibrosis patients^11^	Bosso et al	CFP + ATR	More frequent synergy in the combination group than with cefepime plus ciprofloxacin
Dual β-lactam combination therapy for multidrug resistant *Pseudomonas aeruginosa* infection: enhanced efficacy in vivo and comparison with monotherapies of penicillin-binding protein inhibition^14^	Siriyong et al	AMP, MRP, PIP, ATR, CFX, CTX, CDX, and CFZ (total of 28 combinations)	MRP + CTZ, ATR + CTX, MRP + PIP, and ATR + MRP were the combinations with higher synergy. Highest synergy for ATR + MRP
Contemporary in vitro synergy rates for aztreonam combined with newer fluoroquinolones and beta-lactams tested against gram-negative bacilli^12^	Sader et al	ATR + CFZ	Higher synergy between combination of ATR plus other beta-lactams compared with combinations between ATR and fluoroquinolones Highest synergy for ATR + IMI
		ATR + CFP	
		ATR + IMI	
Activity of mecillinam alone and in combination with other beta-lactam antibiotics^17^	Fass et al.	ATR, MLM, CFM, and TIC (total of 6 combinations)	Low synergy against gram-positives
			MLM had marked synergy with CFM and TIC
			Antagonism between CXT and TIC or CFM
Mecillinam alone and in combination with ampicillin or moxalactam in experimental *Escherichia coli* meningitis^19^	Schaad et al	MLM + AMP	Better microbiological outcomes between MLM + AMP and monotherapy against MLM
		MLM + MXL	Superiority of MLM + MXL not proven
Efficacy of dual carbapenem treatment in a murine sepsis model of infection due to carbapenemase-producing *Acinetobacter baumannii*^27^	Cebrero-Cangueiro et al	IMI + MRP	Better outcomes compared with monotherapy
In vitro synergy of β-lactam combinations against KPC-producing *Klebsiella pneumoniae* strains^28^	Lawandi et al	IMI or MRP + PIP/TAZ IMI or MRP + ERT IMI or MRP + CTZ	MRP + PIP/TAZ with the highest synergy
Efficacy of ampicillin plus ceftriaxone in treatment of experimental endocarditis due to *Enterococcus faecalis* strains highly resistant to aminoglycosides^33^	Gavaldà et al	AMP + CTR	Synergy between the two beta-lactams and better imagiologic outcomes (lower vegetations)

AMP = ampicillin; ATR = aztreonam; CDX = cefadroxil; CFM = cefamandol; CFP = cefepime; CFX = cefuroxime; CFZ = ceftazidime; CTR = ceftriaxone; CTX = cefotaxime; CTZ/AVI = ceftolozan/avibactam; CXT = cefoxitine; CZN = cefazoline; ERT = ertapenem; IMI = imipenem; MLM = mecillinam; MRP = meropenem; MXL = moxalactam; PIP = piperacillin; PIP/TAZ = piperacillin/tazobactam; TIC = ticarcillin.

Antimicrobial resistance and the lack of viable options could relight the enthusiasm over dual beta-lactam therapy. However, it may not be suitable in all the situations and should be tailored to each individual case to choose the optimal balance between efficacy and safety for patients.

This review intends to identify in which cases dual therapy could be used and why it is not a panacea.

## Materials and methods

### Data sources

An exhaustive literature search was performed using PubMed as the main database up to December 10, 2020. An additional internet search was conducted to find unpublished scientific material.

### Search strategies

The most relevant search terms were the following: “double beta-lactam,” “dual beta-lactam,” and “beta-lactam combination.” References cited by these publications identified from this search strategy, relevant reviews, and forward citations were further evaluated. In relevance to other topics we further discuss throughout this review, we have also used other search terms such as “*Pseudomonas aeruginosa*,” “*Enterococcus*,” “*Staphylococcus aureus*,” “*Enterobacteriaceae*,” and “carbapenemase-producing bacteria” linked between each other by “OR” and linked to the previous group of search terms by “AND.” Other relevant search terms were “side effects,” “adverse events,” and “microbiome.” In most of the search entries, the option “best match” was activated.

### Inclusion criteria

We included articles pointing to in vitro or in vivo information in the form of case reports, reviews, or clinical trials. No restrictions were imposed on the patient population, infection type, length of follow-up, or the specific beta-lactams combined.

### Exclusion criteria

We excluded all the articles not written in English language, those published before 1975, and those with incomplete or inaccessible data.


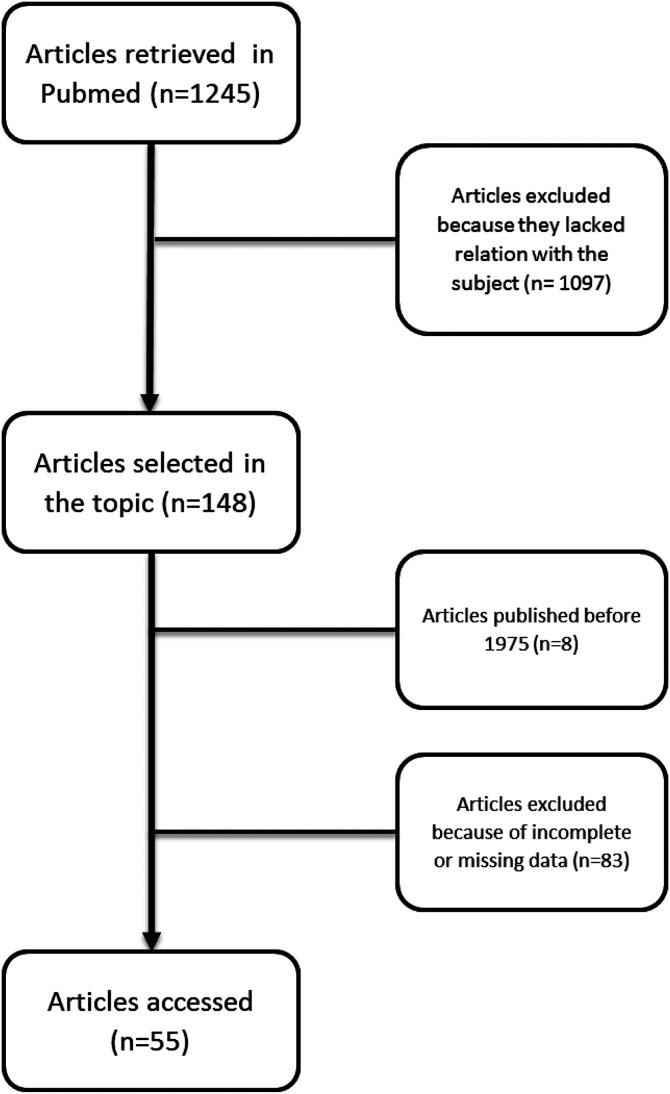


## Dual beta-lactam therapy: Does it work?

According to the last International Guidelines for the Management of Sepsis and Sepsis Shock,^3^ one should initiate the correct antimicrobial, intravenously, within the first hour after the diagnosis for both sepsis and septic shock. This recommendation is based on the increase in mortality seen for every hour delay of antibiotics administration. In these circumstances, dual therapy could broad the spectrum, assuring adequate coverage of the potential pathogens and thereby improving the patient's outcome.

The interest of the scientific community in combining different antibiotics started in the 1950s when the high incidence of relapse of endocarditis treated with penicillin G alone was largely reduced by simply adding streptomycin.^4^ Since then, the concepts of *synergy* and *antagonism* have sparked the curiosity of microbiologists, biochemists, and molecular biologists who began testing combinations of a wide range of antibiotics available at that time. In summary, the *theory of synergy* states that the effect of certain antibiotics used together is more potent than the sum of their individual effects. *Antagonism*, on the other hand, occurs when the combined effect of antimicrobial drugs is less than the sum of the independent effects measured separately.

All beta-lactams covalently bind to and, thereby, are responsible for the inactivation of one or more penicillin-binding proteins (PBPs). Nonetheless, they differ from each other according to their PBP-binding patterns. Using a combination of 2 beta-lactams potentially enables the inactivation of various PBPs simultaneously, thus reaching the *synergistic effect*, increasing their bactericidal activity, and preventing the occurrence of newly developed resistant bacteria.^5^

## Empirical therapy

Beta-lactams represent the largest and most studied class of antimicrobials. In recent decades, we have witnessed an increase in data about their pharmacokinetics and pharmacodynamics. There is also a considerable amount of data regarding their safety profile and in this way appears to be a well-suited class for dual empiric therapy.

Most studies dedicated to the role of dual beta-lactam as an empirical therapy were performed in neutropenic populations. In 2019, Jiao et al^5^ reviewed 13 randomized controlled trials reported between 1972 and 1993. They concluded that empirical and nonoptimized dual beta-lactam therapy in patients with febrile neutropenia achieved similar clinical and microbiological outcomes compared with the other usual combination (beta-lactam plus an aminoglycoside) and also had significantly better safety compared with the latter.

However, some of the studies performed at the time used multiple daily doses of aminoglycosides, in contrast with the single daily dose used today which is known to be much less nephrotoxic. Despite having been chosen empirically, at least one of the beta-lactams in the combination had an antipseudomonal activity.

In non-neutropenic patients, File and Tan^6^ published the results of a prospective controlled clinical trial in 1983 in which 41 patients with soft-tissue infections were distributed in 2 groups: 21 patients treated with cefoxitin alone and the remaining 20 were treated with a combination of cefoxitin and mecillinam. In this study, the authors compared the efficacy and safety of the two antibiotic schemes. The overall response and survival rates were similar between the two groups, but the microbiological response was better in the combination group, although this difference was not statistically significant. The rates of therapeutic failures and safety issues were also equivalent in both groups.

Seven years later, Menichetti et al^7^ published a prospective randomized trial comparing two beta-lactam schemes exclusively in febrile cancer patients with adequate neutrophil counts (defined as >1000 neutrophils/mm^3^). The aim of this study was to compare the outcomes of febrile patients treated by ceftriaxone alone and patients treated by a dual beta-lactam combination consisting of cefazolin and aztreonam. Overall, 154 febrile patients were randomized into the two groups. None of the patients had undergone recent chemotherapy nor received prophylactic antibiotics. The overall response was similar between the two groups. Outcomes of patients with microbiologically documented infections were slightly better in the dual beta-lactam group (both regarding gram-negative and gram-positive microorganisms). By contrast, those patients with just clinically documented infections had better outcomes in the ceftriaxone group. However, none of these differences were statistically significant.

## Dual therapy in documented infections

### Pseudomonas aeruginosa

*Pseudomonas aeruginosa* is a ubiquitous, nonfermenting gram-negative rod that belongs to the Pseudomonaceae family. It can be isolated from various living sources constituting, seldom, a member of the normal human flora. It has a tremendous capacity to thrive in places with very little amount of nutrients and tolerates a variety of physical conditions. In this way, *P aeruginosa* can be found both in the community and in the hospital, being an important organism responsible for nosocomial infections such as hospital-acquired pneumonia, urinary tract infections, and bacteremia.

Treating *Pseudomonas* infections represents a therapeutic challenge in both the community-acquired and hospital-acquired settings because of its ability to develop resistance to multiple classes of antimicrobial drugs, even during the course of treatment. Infections caused by drug-resistant *P aeruginosa* are linked to the increase of morbimortality, length of hospital stay, need for surgery, and overall cost of treatment.^8^

In vitro studies performed in the 1980s comparing the synergy effect of multiple drug combinations for inhibiting *P aeruginosa* growth have found synergy between various beta-lactams, such as moxalactam and ceftazidime, and piperacillin or ticarcillin. However, their synergistic effect fell behind the one seen in combinations using beta-lactams and aminoglycosides.^9^

Binding to a specific *Pseudomonas* PBP named “PBP4” was shown to largely upregulate the chromosomal AmpC beta-lactamase.^10^ This PBP is the highest affinity target of molecules such as carbapenems and cefoxitin. Other drugs, such as ticarcillin and piperacillin, extensively used in *Pseudomonas* infections, are subject to inactivation by the AmpC beta-lactamase. Therefore, to minimize the clinical impact of resistance due to this enzyme, it would be logical to create a dual beta-lactam combination composed of at least one molecule that is not inactivated by it.^5^

Other resistance-conferring mutations are those that lead to upregulation of drug efflux pumps or the inactivation of the porin *OprD*, decreasing the drug permeability of the bacterial membrane. Finally, *P aeruginosa* resistance to multiple drugs can also result from the acquisition of transferable resistance genes such as those encoding extended-spectrum beta-lactamases or carbapenemases.

Bosso et al,^11^ in 1991, found in vitro synergy against *Pseudomonas* strains when cefepime was combined with aztreonam, totalizing 44% of the patients involved vs. the 29% found with the cefepime plus ciprofloxacin combination. Later studies conducted by Sader et al^12^ and Song et al^13^ confirmed this synergistic effect against *Pseudomonas* when combining aztreonam with cefepime or ceftazidime.

Siriyong et al^14^ tested the efficacy of dual beta-lactam therapy against multidrug-resistant *P aeruginosa* infections using a larvae species (*Galleria mellonella*) as an infection model. In this study, a group of 8 beta-lactam drugs (ampicillin, meropenem, piperacillin, aztreonam, cefuroxime, cefotaxime, cefadroxil, and ceftazidime) were tested in both monotherapy and dual therapy (for a total of 28 combinations). Piperacillin, aztreonam, ceftazidime, and meropenem were the top 4 drugs with the greatest therapeutic value when used in monotherapy. Many of the dual therapeutic combinations showed improved survival when compared with their monotherapy counterparts. The top four combinations were meropenem plus ceftazidime, aztreonam plus cefotaxime, meropenem plus piperacillin, and aztreonam plus meropenem. These four combinations were also tested for survival of the larvae with increasing *inoculum*. Aztreonam plus meropenem was the only one which still kept some activity after the larvae were exposed to the highest *inoculum*. Interestingly, the enhanced efficacy of meropenem plus ceftazidime and aztreonam plus meropenem combinations could not be explained by increased inhibition of a wider range of PBPs. One possible explanation for this observed effect was the stimulation of the larvae's innate immune system by exposing it to various antibiotics.^13^

A meta-analysis based on 13 randomized controlled clinical trials found no statistically significant difference in both the clinical and microbiological responses between dual beta-lactam therapy and a beta-lactam plus an aminoglycoside regarding *Pseudomonas* infections.^12^

### Enterobacteriaceae

*Enterobacteriaceae* are a large and heterogeneous group of gram-negative rods which usually colonize the gut of mammals. Some examples belong to species of *Escherichia*, *Citrobacter*, *Enterobacter*, *Proteus*, *Hafnia*, and *Klebsiella*. This class represents a wide range of bacteria, from beneficial commensal microbiota to opportunistic pathogens in immunocompromised hosts, and some may even cause serious disease in otherwise healthy individuals.^15^

Much like *P aeruginosa*, most studies about the effect of dual beta-lactam combinations in treating infections by *Enterobacteriaceae* used molecules that are no longer available. In addition, the synergistic effect occurred more frequently when the monobactam aztreonam or a third-generation cephalosporin was incorporated into the combination.^16^

In 2003, Sader et al^12^ performed a study to compare the in vitro effect of various antibiotic combinations, namely aztreonam combined with other beta-lactams (cefepime, ceftazidime, and imipenem) against *Enterobacteriaceae* species. Twelve species were studied, including *Citrobacter freundii*, *Enterobacter aerogenes* (recently renamed *Klebsiella aerogenes*), *Escherichia coli*, *Enterobacter cloacae*, *Hafnia alvei*, *Klebsiella pneumoniae*, *Klebsiella oxytoca*, *Morganella morganii*, *Pantoea agglomerans*, *Proteus mirabilis*, *Providencia stuartii*, and *Serratia marcescens*. They found that the percentage of synergistic or partially synergistic interactions was higher among aztreonam/beta-lactam combinations when compared with aztreonam/fluoroquinolone. Among the beta-lactam combinations tested, aztreonam/imipenem shown the best results, with a synergistic effect against 32.5% of strains and partial synergy against 55% of them.

Fass^17^ was another author who studied the dual-beta-lactam combinations in the early 1980s and, mainly, to the importance of mecillinam in those combinations. For instance, he showed a potent synergistic effect against all *Enterobacteriaceae* tested when mecillinam was combined with ticarcillin and cefamandole (a second-generation cephalosporin). He and his colleagues also looked for the role of the combination of the acylated form of ampicillin (azlocillin) and cefotaxime against *Enterobacteriaceae* species, confirming the existence of a wide range of synergistic effect of these two molecules against these bacteria. However, there also seemed to be a variable degree of antagonism in approximately 11% of *Enterobacteriaceae*.^18^

There were also encouraging results in in vivo studies. In 1982, Schaad et al conducted a study in an *E. coli* rabbit meningitis model. One of their objectives was to compare the bacteriologic efficacy of mecillinam, either alone or in combination with ampicillin and moxalactam. The reduction in cerebrospinal fluid bacterial concentrations observed with the combination of ampicillin and mecillinam was significantly greater (*P* < .01) than that found with either mecillinam or ampicillin alone. However, there were no significant differences between the bactericidal effect of moxalactam alone and the combination with mecillinam.^19^

### Carbapenemase-producing bacteria

There are currently no optimal treatment regimens to face the rapid rise of carbapenemase-producing bacteria. Once again, beta-lactam combinations may be a partial solution to this problem.

Pioneer studies using animal models of infections and case reports of salvage treatments of patients have supported the use of dual-carbapenem combinations for treating infections by carbapenemase producers of both classes A and B of Ambler classification.^20–22^ Recently, there have been also in vitro studies suggesting this carbapenem combination might also be useful against the peculiar class D. In one of those articles, synergy between imipenem and meropenem was found in 25% of the isolates carrying *OXA-*like penicilinases.^23^

The rationale for this antibiotic strategy would be that one of the carbapenems used (particularly ertapenem) may bind to the active site of the carbapenemase with higher affinity, in this way preventing the hydrolysis of the other carbapenem molecule. The efficacy of these combinations is variable and depends on the type of bacteria and molecules used. For example, those containing imipenem or doripenem seem to be more effective against strains of *Klebsiella pneumoniae*.^24,25^

In 2017, in a two-center, observational, case–control study including critically ill patients infected with carbapenem-resistant *K pneumoniae*, De Pascale^26^ reported significantly higher mortality rates for patients treated with “standard therapy” (ie, tigecyclin, gentamicin, or colistin) compared with those treated by a dual-carbapenem strategy.

Cebrero-Cangueiro et al^27^ also tested this in a murine sepsis model of infection because of *OXA*-producing *Acinetobacter baumannii* resistant to both imipenem and meropenem. In this trial, there were two different strains of *A baumannii*: one producing *OXA-23* and the other producing *OXA-58*. They compared the outcomes of the mice that were given this combination with those of the mice that were given the same antibiotics in monotherapy. They found that both meropenem and its combination with imipenem resulted in lower mortality and improved bacterial clearance from spleen compared with both the control and imipenem-only groups. Meropenem alone and the carbapenem combination significantly decreased the blood *OXA-58*–producing bacterial load compared with the other groups. Finally, the blood load of *OXA-23* producer was significantly decreased by the combination of carbapenems compared with the effect of meropenem monotherapy.

More recently, Lawandi et al^28^ performed time-kill assays on 24 unique KPC-producing *K pneumoniae* using combinations including meropenem or imipenem and one of the following: ertapenem, piperacillin/tazobactam, or ceftolozane/tazobactam. This in vitro analysis showed that the combination of piperacillin/tazobactam with meropenem was synergistic against 70.8% of the isolates, followed by ertapenem with meropenem (58.3%) and ceftolozane/tazobactam with meropenem (41.7%).

All these data support the theory of dual-carbapenem synergy and raise the possibility of using this option for treating severe infections by carbapenemase-producing microorganisms, especially when other therapies are unavailable or prohibitively toxic.

### Enterococcus

The genus *Enterococcus* comprises a group of gram-positive bacteria of great importance for their role as major causative agents of healthcare-associated infections. *Enterococci* are resilient and versatile bacteria capable of surviving under very challenging conditions and hence adapt very easily to the healthcare environment. The two species responsible for most enterococcal infections are *Enterococcus faecalis* and *Enterococcus faecium*. They both demonstrate natural resistances to commonly used antibiotics such as cephalosporins, aminoglycosides, cotrimoxazole, and clindamycin.^29^ In addition, the rising resistance to vancomycin seen in this genus further complicates detreatment of infections caused by these organisms.

*Enterococcus* is the third cause of endocarditis worldwide and often affects the elderly. The standard treatment of enterococcal endocarditis has been the combination of penicillin G and gentamicin since 1984.^30^ However, owing to the rising concerns of aminoglycosides nephrotoxic potential with prolonged use, researches have been trying to figure out a safer alternative without decreasing efficacy. In addition, the rising prevalence of highly aminoglycoside-resistant *Enterococcus* could potentially turn this into a nonviable combination.

Dual beta-lactam combinations have been suggested as an alternative therapy. The research for this alternative started back in 1995 with Mainardi et al,^31^ who were the first describing the synergy effect between amoxicillin and cefotaxime. The results of their trial showed a decrease in the minimum inhibitory concentration of amoxicillin in the presence of cefotaxime and in the same way with cefotaxime in the presence of amoxicillin. Four years later, Gavaldà et al^32^ using a rabbit *E. faecalis* endocarditis model also discovered that rabbits treated with ampicillin and ceftriaxone had lower vegetation counts than those treated with just ampicillin. The same authors conducted another study in rabbits comparing the combinations ampicillin plus ceftriaxone and ampicillin plus gentamicin. They concluded that the two regimens were comparable in efficacy, and therefore, ampicillin plus gentamicin could be an alternative to the standard treatment at the time.^33^ This in vitro synergistic effect of the association of ampicillin with a beta-lactam was subsequently confirmed for a wider range of other beta-lactams, including cefazolin, cefepime, ceftobiprole, and ceftaroline.^34^

Recall that although *Enterococcus* is intrinsically resistant to cephalosporins, the association between cefotaxime/ceftriaxone and ampicillin results in binding and inactivation of a broader range of PBPs, leading to enhanced activity than either agent alone.^33^

Then came two important clinical trials. The first one, a multicenter, observational, and open-labeled study also conducted by Gavaldà et al,^35^ tested and confirmed the safety of the ceftriaxone-ampicillin combination for 43 patients with endocarditis because of *E faecalis*. More recently, Fernández-Hidalgo et al^36^ directed a multicenter, nonrandomized cohort study comparing the safety and efficacy between the two antibiotic regimens: ceftriaxone plus ampicillin and ampicillin plus gentamicin in 246 patients. There was no difference in mortality while on treatment and during the 3-month follow-up period. In addition, there were no differences in rate of relapse or treatment failures. However, patients treated with ampicillin plus gentamicin had significantly more side effects requiring therapy withdrawal. However, they only tested long treatment durations with gentamicin and not the 2-week long treatment that is now known to be safer.

Following the favorable results in these human cohort studies, the combination of ampicillin plus ceftriaxone was included in the latest guidelines of the European Society of Cardiology as a treatment of first choice.^37^ However, despite the success of this regimen in endocarditis, we did not find any study regarding its use in other clinical situations.

### Staphylococcus aureus

Bacteria of the genus *Staphylococcus* are gram-positive cocci that are usually microscopically observed as grape-like clusters. They are nonmotile, non–spore-forming, and catalase-positive bacteria. Because of a bound coagulase and its extracellular staphylocoagulase, *Staphylococcus aureus* has the ability to clot plasma, therefore distinguishing it from other *Staphylococcus* species.

*S aureus* is ubiquitous and may be part of the normal flora from the inguinal, axillae, perineal, and inguinal areas. It is also estimated that this species colonizes the anterior nares of 20%–80% of the human population.

*S aureus* is one of the leading causes of multiple community-acquired and hospital-acquired bacterial infections. *S aureus* is also one of the most frequent causes of bacteremia, carrying the highest mortality^38^ and accounting for approximately 20% of all blood stream infections.^39^

Despite infections with methicillin-susceptible (MSSA) and methicillin-resistant *S aureus* (MRSA) being both linked to high mortality rates, infections due to MRSA lead to worse outcomes showing an overall mortality as high as 25%–50%.^39^ Vancomycin is still the most frequently chosen drug to treat infections because of this microorganism. In the past years, new drugs such as linezolid, ceftaroline, and daptomycin have emerged and are being increasingly used by the healthcare providers but, unfortunately, not without a few disadvantages. Despite MRSA being inherently resistant to beta-lactams, treatment with a beta-lactam combination might be an alternative for treating infections due to MSSA.

Management of MSSA persistent bacteremia comprises surgical source control and early start of antistaphylococci beta-lactams (oxacillin, flucloxacillin, and cefazoline being the most commonly used). Owing to the high efficacy of these drugs, there are only few data about combination therapy. However, there are in vitro data that support the existence of a synergistic effect between cefazolin and ertapenem, and there are case reports demonstrating its success in for patients with persistent *S aureus* bacteremia.^40^ More recently, Ulloa et al^41^ published a series of 11 cases treated successfully with ertapenem and cefazolin as salvage therapy. They described a rapid clearance of bacteremia, with 88% of cases having negative hemocultures after 24 hours of treatment, who were otherwise failing with standard therapy. They state that the clearing of bacteremia after 24 hours of treatment with vegetations measuring over 2 cm “far exceeds the predicted expectations from the in vitro studies.” The authors stipulate that there could be a “*sensitization of MSSA*” exposed to these two antibiotics to the innate immune system. Another possible reason could be a powerful PBP-1 interference in response to the addition of ertapenem.

Clinical trials testing this combination are however still lacking.

## Why shouldn’t we do it?

### Potential antagonism between some of the molecules

During our research, we found a significant number of studies documenting potential antagonism between beta-lactam molecules.^19,42-45^ Unfortunately, these were all performed in late 1970s and 1980s. This phenomenon has been explored with different strains, and it appeared that against strains of *Escherichia coli*, *K pneumoniae*, *Proteus mirabilis*, *Enterococcus* spp, and *Staphylococcus* spp, the combinations of beta-lactam molecules exhibited no antagonism effects. On the other hand, a high incidence of antagonism was observed in strains which produce an inducible chromosome-encoded cephalosporinase (*P aeruginosa*, *Serratia* spp, *Enterobacter* spp, and other *Proteus* spp), particularly with combinations containing cefoxitin.^46^

Several mechanisms have been hypothesized to account for the antagonistic interference between two beta-lactams in vitro.^4^,^47^

According to Acar et al,^42^ there is a possibility that one of the drugs may induce beta-lactamases. The synthesis of chromosomally mediated beta-lactamases can be induced by almost every beta-lactam substrate.^43^ Observations suggest that for each beta-lactam, there is a concentration at which induction of beta-lactamase is optimum.^48,49^ However, antagonism is mainly observed when cefoxitin, imipenem, or clavulanic acid is combined with penicillins or cephalosporins.^50,51^ Interestingly, with sulbactam and tazobactam, antagonism activity was not observed.^52,53^ The beta-lactamase inducibility could be based on the structure-activity relationships. Indeed, Kanazawa et al^54^ clarified that a group on the C-2 side chain in the carbapenem skeleton influences the antagonistic effect of carbapenems.

Another hypothesis is that the antagonist molecule could combine with the beta-lactamase, increasing its hydrolysis efficacy. The antagonist could also compete with the most effect molecule, preventing it from reaching the binding site to the bacteria that is critical for the bactericidal effect to occur. In *P aeruginosa*, antagonism could be linked to porin selection. Entry of the most active molecule could be blocked by the other beta-lactam.^55^ Finally, Acar et al^42^ stipulated that the antagonist could induce a biological change to the bacterial surface, thereby stopping the attachment of the most effective drug to its binding site.

The clinical relevance of antagonism remains a controversial subject because controlled studies were so rare to support the sporadic cases in physicians' personal experience.

Kuck et al^46^ noticed that these in vitro effects were predictive of in vivo responses in mice.

In their study, mice were infected intraperitoneally with different *Enterobacteriaceae* and *Pseudomonas*. Cephalosporin (cefoxitin, cefamandole, and cephalothin) was administered immediately before the penicillin antibiotic. They observed that the Effective Dose 50% (ED_50_) of piperacillin was increased four-fold to eight-fold for the *Pseudomonas* infection when treatment included cefoxitin and two-fold to four-fold for the *Enterobacter* infection.^47^ In another study of intraperitoneal infection, this time assessing the interaction between cefamandole or carbenicillin and cefoxitin, mice were infected with *Enterobacter cloacae* and *P aeruginosa*. Bacteria were recovered from the heart of nonsurviving animals and tested for their susceptibility to the protective drug used in treatment. In the same way as the previous study, the antagonistic effect of cefoxitin on cefamandole was reflected by a lack of well-defined reductions in bacterial blood counts after cefamandole administration.^56^

The clinical significance of these antagonisms is still unknown. Because of this potential for antagonism, the combination of clavulanic acid, cefoxin, or imipenem with other beta-lactam antibiotics should be considered carefully for use against bacterial strains possessing inducible beta-lactamases.

## Increased side effects

### Alteration of microbiome

The human intestine has a high density and variety of microbes that are crucial to human physiology and nutrition. Furthermore, intestinal bacteria have a 25-fold higher rate of gene transfer than bacteria in other settings, and antibiotic exposure increases the horizontal transfer even further.^57,58^

Antibiotic usage, in this way, leads to a disruption of the microbiome leading to changes in the abundance of certain genera, increase in the colonization with potentially pathogenic (p.e. *Enterobacter*) or opportunistic (p.e. *Clostridioides*, *Candida spp*.) microorganisms, as well as the development of antibiotic resistance.^48^ Ultimately, perturbations of the intestinal flora could favor a wide range of diseases from mild diarrhea to life-threatening toxic megacolon. In addition, many studies show that antibiotics increase the abundance of multidrug-resistant gram-negative microorganisms that, in turn, might make patients more prone to infections by antibiotic-resistant bacteria.^59^

A large review was conducted^48^ at the end of 2019 aiming to summarize studies that have investigated the effect of antibiotics on the composition of the human microbiome. They concluded that penicillin only had minor effects on the abundance in different taxa in the microbiome and did not increase drug resistance. Some cephalosporines and carbapenems did not have a great effect on resistance increase either. Amoxicillin/clavulanate decreased bacterial diversity, increasing the abundance *Enterobactereaceae* (mainly *Citrobacter spp.*, *Enterobacter spp.*, *and Klebsiella*). Amoxicillin alone was also responsible for the increase of the *Enterobactereaceae* with the exception of *E coli* (along with cephalosporins, it decreased the *E coli population*). Carbapenems, amoxicillin, piperacillin, ticarcillin, and cephalosporins (except fifth-generation cephalosporins) were responsible for the increased abundance of *Enterococcus* spp. Piperacillin, ticarcillin, and carbapenems strongly decreased the abundance of anaerobic bacteria. Amoxicillin and cephalosporins were the beta-lactams more frequently associated with newly acquired or increased colonization by *Clostridioides* (formerly known as “*Clostridium*”).

Importantly, amoxicillin, amoxicillin/clavulanate, cephalosporins, and carbapenems increased the abundance of yeast. The use of amoxicillin also associated with a decrease of *Blautia spp*^60^ and *C spp*^50^ in the bowel, considered to have anti-inflammatory properties and whose lack has been linked to colon cancer^61^ and irritable bowel syndrome,^62^ respectively.

This variability of influence in the microbiome according to specific molecules was consistent across various studies. However, the authors also observed that the antimicrobial influence in the microbiome also depends on several factors such as drug dose and formulation as well as treatment duration. Another important factor would be the specific pharmacodynamics of the antibiotic: For instance, the influence of ceftriaxone has a lot to do with its known biliary clearance.

The authors also reviewed ten studies that included 210 participants and 40 controls that investigated the effect of a combination of antibiotics. Unfortunately, none of them referred to beta-lactam combinations, and therefore, we do not know the exact influence of a dual beta-lactam therapy on the microbiome.

Along with this information, we have also found an article by de Man et al,^63^ conducted in two identical neonatal intensive care units. They observed an increased development of resistance with empiric use of intravenous amoxicillin plus cefotaxime compared with penicillin G and tobramycin or flucloxacillin and tobramycin.

### Safety issues

Beta-lactams are the most commonly prescribed antibiotics in both the hospital and community settings. Despite remaining one of the safest antimicrobial classes today, they still carry some adverse effects that all the clinicians should be aware of.^64^

Afar from the possibility of disrupting the normal bowel flora and increasing the risk for infection by *Clostridioides* that we already spoke about, side effects from beta-lactams, although rare, can attain the hematological, renal, and neurologic systems. Nevertheless, the most frequent reactions are allergic ones, although they are still rare.^65^

Clinical comparative studies to verify the safety of dual beta-lactam strategy are still lacking, and most of the studies date several decades ago. Like we already said before, they used outdated molecules and doses, and thus, we need to be careful when interpreting their conclusions. For instance, the high reported incidence of phlebitis could be explained by the early and low-quality injection formulations. Without forgetting this caveat, a recent review conducted by Jiao et al^5^ comparing the safety between dual beta-lactam regimen and the combination of beta-lactam plus an aminoglycoside observed a significantly lower oto and nephrotoxicity caused by the beta-lactam combination, as expected. They also reported high incidences of coagulation, hypokalemia, and phlebitis with both regimens. The frequency of hypokalemia, particularly, was significantly higher compared with the beta-lactam/aminoglycoside association.

## Discussion

Overall, all these data seem to support the use of dual beta-lactam therapy. In the particular case of endocarditis, beta lactam combinations seem to be safer and as effective as antibiotic combinations containing aminoglycosides (Table 1).

In addition, antibiotics such as aztreonam and mecillinam, not that used in a global perspective, had high synergy with many other beta-lactams. However, the studies were, mainly, in vitro, and so clinical reports and randomized control trials are lacking to prove whether this synergistic effect remains true in vivo.

Although less information is available, combination between aztreonam and carbapenems also seemed to be a great alternative regimen to face infections against MDR *Enterobactereaceae*.

Nevertheless, most information regarding clinical trials dated from the 80s/90s and some of the most recent drugs such as fifth-generation cephalosporins were not featured. In addition, some of the doses and timing of administration of some molecules were outdated, and because of that, we should be aware about drawing rushed conclusions. Some of the most interesting trials on animal models such as the apparent success of the combination of aztreonam and meropenem against MDR *Pseudomonas* need to make the jump into clinical trials because it is an area we do not have that much of choices available.

Other area that lacks data is the dual therapy in *E faecalis* because, to our knowledge, it has only been tested for endocarditis. However, considering *E faecalis* also an agent that attains both the urinary and abdominal systems, it would be of major importance to test this dual therapy in those kinds of infection.

Antibiotic antagonism, the impact on gut microbiota and increasing side effects all contribute to physicians hesitancy on choosing dual beta lactam strategy. Cefoxitin stands out as the main beta-lactamase inducer, favoring antagonism when combined with many beta-lactams. The effect of beta-lactam combinations on the microbiome is still a mystery because we found only one paper comparing the emergence of resistances between only a few beta-lactam combinations. Nevertheless, the effects of antibiotics on the gut microbiota likely depend on the spectrum of activity and elimination pathways. Because one of the advantages of this dual therapy, as stated earlier, is to broaden the spectrum, one might hypothesize for a greater influence on the microbiome by using this regimen. Last but not least, despite beta-lactams being one of the safer classes of drugs, there are no data evaluating the dual therapy side effects. For instance, could it be possible that, similar to the mutual potentiation of their antimicrobial effects, one could also expect a potentiation of their side effects? Hypersensitivity reactions hyperbolized because of immunological cascade crossed reaction between the two molecules. Additional studies are needed to answer these questions.
